# The association between COVID-19 preventive strategies, virtual reality exercise, use of fitness apps, physical, and psychological health: testing a structural equation moderation model

**DOI:** 10.3389/fpubh.2023.1170645

**Published:** 2023-07-06

**Authors:** Rashid Menhas, Luo Qin, Zulkaif Ahmed Saqib, Muhammad Younas

**Affiliations:** ^1^International Institutes of Medicine, The Fourth Affiliated Hospital Zhejiang University School of Medicine, Yiwu, Zhejiang, China; ^2^College of Urban Transportation and Logistics, Shenzhen Technology University, Shenzhen, China; ^3^School of Education, Soochow University, Suzhou, Jiangsu, China

**Keywords:** fitness apps, virtual reality exercise, psychological health, physical health, Chinese society

## Abstract

**Background:**

Directly or indirectly, individual psychosocial characteristics, motivation, and health consciousness factors help to maintain physical and psychological health through maintaining physical activity.

**Objective:**

In the current study, we investigated how fitness apps moderate the links among COVID-19 preventive strategies, virtual reality exercise, physical health, and psychological health in Chinese society.

**Method:**

A nationwide online survey across China was conducted under a snowball sampling design from February to June 2022. A total of 3,000 questionnaires were distributed across China via online platforms. A total of 2,795 complete detailed replies were included in the final analysis. Structural equation modeling techniques were employed to analyze the collected data through Smart-PLS 3.0.

**Results:**

It has been statically proved that all the scales used in this inquiry to determine the mean scores, standard deviation, excess kurtosis, and skewness values were reliable and produced satisfactory results. The overall results (H1: *β* = 0.385, *t* = 15.699, *p* = 0.000; H2: *β* = 0.159, *t* = 7.405, *p* = 0.000; H3: *β* = 0.122, *t* = 5.435, *p* = 0.000; H4: *β* = 0.143, *t* = 6.493, *p* = 0.000; H5: *β* = 0.157, *t* = 6.444, *p* = 0.000; H6: *β* = 0.184, *t* = 9.071, *p* = 0.000; H7: *β* = 0.192, *t* = 9.319, *p* = 0.000; H8: *β* = 0.235, *t* = 11.899, *p* = 0.000; H9: *β* = −0.114, *t* = 4.872, *p* = 0.000; H10: *β* = 0.042, *t* = 2.872, *p* = 0.004; H11: *β* = 0.041, *t* = 2.699, *p* = 0.007) supported our hypothetical model and explained that using fitness apps and virtual reality exercise benefits physical and psychological health.

**Conclusion:**

The fitness app’s primary purpose during and after the pandemic is to motivate users to keep up with their regular at-home workouts. Exercise and active living are helpful in the prevention of risk factors associated with physical inactivity.

## Introduction

An unprecedented amount of internet and digital technology usage has resulted from COVID-19-related actions, including self-quarantine and locking down businesses, schools, and leisure venues. In the COVID-19 age, people are moving almost every element of their everyday lives to cyberspace at an accelerated rate. However, it is still unknown if and how using the internet might worsen or improve the psychological anguish brought on by COVID-19 (1). Numerous research studies have shown that the COVID-19-related restriction measures affected lifestyle behaviors, such as playing sports and engaging in physical activity (PA), and disturbing daily routines and PA (2). Health professionals often employ mobile health (mHealth) applications to promote behavioral health outcomes and enhance users’ health. On smartphones and other mobile devices, mHealth software improves health and avoids medical disorders among the general population (3).

Physical activity and exercise are vital functions in a person’s life. Long-term advantages include bettering one’s overall physical and mental well-being. Additionally, it helps lower the chances of several illnesses, including type 2 diabetes, cancer, and cardiovascular disease, which are all reduced in those who regularly engage in physical activity. Health and wellness have undergone significant changes due to exercise and health applications. During the COVID-19 shutdown, public areas, including gyms, squares, parks, and walking paths, were shuttered. Physical exercise may naturally maintain the immune system (4). Muscular atrophy, low lung capacity, increased frailty risk, and mental health problems are all factors that may be mitigated via regular exercise and other forms of physical activity. COVID-19 may trigger an immunological response that damages cells and tissues by releasing excessive immune cells and cytokines (5). With cutting-edge capabilities like an integrated accelerometer, global location, internet connection, smart apps, and digital platforms provide a beneficial physical activity intervention for health and well-being (6). Disease management apps and health behavior change applications are two categories of innovative health and fitness applications. Smart systems that control a patient’s medicine and guard against problems are known as disease management apps. These applications are designed to examine patient data and encourage self-care. On the other hand, the application that supports promoting physical activity and fitness for people of all ages is behavioral change in health (7).

The advancement of technology has resulted in a continuous transformation of our homes and daily routines. About twenty years ago, health wearable’s (such as pedometers) that assessed physical activity and monitored and analyzed human movement patterns for analysis replaced physical activity surveys (8). The proliferation of health-focused smartphone applications has made it possible to keep tabs on anything from our weight to our blood pressure and overall physical activity levels (9). Accelerometers that distinguish between active and inactive use have just begun appearing in consumer electronics like smartphones and global positioning system (GPS) trackers. Due to scientific advancements, the public may be more likely to engage in sedentary behavior. Still, health professionals can empirically assess physical activity and passive behavior to address many health issues. Current electronic devices appeal to medical professionals because they combine cutting-edge innovations like mobile app software, global positioning system hardware, and wearable health monitors with improving usability, accuracy, and breadth of coverage (i.e., data from multiple sources on a single device). With this data, researchers can better understand physical activity patterns and motivate people to change their activity levels (10). Apps that can be used from a mobile device provide an excellent opportunity to improve people’s home exercise routines by giving them access to health resources whenever needed.

Information and communication technology (ICT) is often utilized regarding instructional methods and physical well-being. As an alternative to standard medical therapy, technologically-based therapies to increase physical activity are available (11). Several Chinese smartphone applications encourage users to exercise, including KEEP, Gu Dong, and Yue Dongquan. Applications promoting physical activity may be loosely classified as fitness or running apps. The trend of using fitness apps and virtual reality exercise significantly increased in China during COVID-19 (12).

Using health apps, users may track their progress or establish goals for their diet or activity. These applications may also be used for weight control, medication adherence, health literacy, and online consultations. Even while mHealth applications are viewed favorably, it is not apparent whether they are essential for users to adopt and maintain long-term healthy behaviors. Prior research primarily focused on the desire to utilize mobile health applications, with little actual user research. As a result, assessing actual use may not depend on one’s willingness to utilize it (13).

This analysis revealed that health behavior change theories and evidence-based information are underrepresented in fitness applications (14). Thus far, studies have looked at how using these applications affects people’s exercise habits. Most research has verified the beneficial influence of fitness apps on exercise (15). When people are motivated to regulate their behavior, they do so under goals distinct from their actions but in line with their core principles and beliefs (16). Evidence from studies grounded in the self-determination theory suggests that a person’s degree of enthusiasm may be used to predict their likelihood of engaging in vigorous physical exercise (17). When a person’s motivation for physical activity is autonomous, they are more likely to practice successful self-regulation (18). The relevance of health behavior theory, data monitoring of health habits, and behavior modification tactics are all vital subjects to learn more about, particularly as innovation permeates more aspects of everyday life (19). It becomes crucial to investigate how fitness apps and virtual reality exercise nexus impact human health’s physical and psychological components. The current study examined how fitness apps moderate the links among COVID-19 preventive strategies, virtual reality exercise, physical health, and psychological health in Chinese society. Many Chinese people, particularly in metropolitan China, are incorporating mobile fitness applications into their regular health and fitness regimens. The widespread use of smartphones, combined with people’s persistent desire to be in shape, has resulted in hundreds of fitness apps that can be downloaded for free or at a nominal fee.

### Theoretical framework

The self-determination theory (SDT), social cognitive theory (SCT), technology adoption model (TAM), and health behavior change model are theories that can be used to explain the relationship between fitness applications, virtual reality exercise, and physical and psychological health. The TAM asserts that a person’s propensity to adopt technology is impacted by how useful and straightforward they believe it to be. In this context, fitness apps and virtual reality exercise can be seen as innovative technologies that can enhance an individual’s exercise experience, increasing their perceived usefulness and ease of use. SDT postulates that people are more likely to engage in and persist in behaviors that align with their intrinsic motivations and needs (20). Fitness apps and virtual reality exercises can provide an engaging and satisfying experience, which can meet individuals’ intrinsic motivations for exercise, leading to an increase in physical activity levels. According to SCT, people’s views of other people’s actions and the social environment’s norms impact those (21). Fitness apps and virtual reality exercises can create a social network and community that promotes physical activity, thereby influencing individuals to engage in physical activity to conform to these norms. According to health behavior change (HBC) theory, a person’s ability to alter behavior is influenced by several variables, including information, attitudes, self-efficacy, and social support (22). Fitness apps and virtual reality exercises can provide individuals with information and feedback about their physical activity, increase their self-efficacy, and provide social support, leading to increased physical activity levels. As suggested by the aforementioned theoretical framework, adopting and using fitness apps and virtual reality exercise can improve physical and psychological health by raising physical activity levels, enhancing physical health and well-being, and offering a pleasurable and engaging experience that can improve psychological well-being. A model was proposed to test the association between COVID-19 preventive Strategies, virtual Reality exercise, use of fitness apps, and physical and psychological health.

### Hypotheses of the study

As people were forced to stay home (23), many turned to VR technology to stay active and healthy. The shift to virtual exercise has provided people an innovative and engaging way to continue their fitness routines, even during a global pandemic. Moreover, VR technology provides users with a more immersive and interactive experience compared to traditional forms of exercise, such as running on a treadmill or lifting weights. Virtual exercise is more appealing and effective for people struggling with traditional methods (24–26). The COVID-19 pandemic has significantly impacted how people exercise, and preventive measures have positively affected the use and popularity of virtual reality exercise. The closure of physical exercise facilities has positively impacted the use and popularity of virtual reality (VR) exercise. COVID-19 preventive strategies may also have indirect health benefits. For example, wearing a mask can help reduce the spread of other respiratory illnesses. So we proposed the following hypotheses according to our proposed model;

*H1*: COVID-19 preventive strategies positive effects on virtual reality exercise.

*H2*: COVID-19 preventive strategies have a positive effect on physical health.

Taking preventive measures can also increase a person’s sense of control and agency in a time of uncertainty and unpredictability. The ability to take action to protect oneself and others can bring a sense of comfort and stability, which can positively impact mental health. Virtual reality exercise directly affects physical health, and using virtual reality technology to engage in physical activity (2) can directly benefit a person’s physical health. Virtual reality can provide an immersive and engaging workout experience, motivating people to be physically active and maintain healthier lifestyles. Virtual reality technology allows users to engage in various physical activities, including virtual sports, dance, and other forms of exercise. Virtual reality exercise (VRE) has direct positive effects on psychological health. It shows that using virtual reality technology to engage in physical activity can immediately benefit a person’s mental well-being (3). Virtual reality can provide an immersive and engaging workout experience, improving mood and reducing symptoms of stress, anxiety, and depression. Physical activity is well-known to affect mental health positively, and virtual reality technology can provide a unique and enjoyable way to engage in physical activity. By creating a fun and interactive environment, virtual reality can help increase motivation and engagement in physical activity, improving mental health (24–26). In addition, virtual reality can also provide a safe and controlled environment for physical activity, which can be beneficial for individuals who may have anxiety or stress related to exercising in public or in front of others. Virtual reality can also provide a convenient and accessible way to engage in physical activity, especially for individuals with limited access to gym equipment or outdoor spaces. It can be significant for individuals who struggle with mental health issues, as regular physical activity is essential for maintaining good mental health. So we proposed the following hypotheses;

*H3*: COVID-19 preventive strategies positively influence psychological health.

*H4*: VRE has direct positive effects on physical health.

*H5*: VRE has immediate positive effects on psychological health.

Fitness apps positively affect physical health, and using fitness applications on smartphones and other mobile devices can positively impact a person’s physical health. Fitness apps offer many features, including workout routines, tracking progress, setting goals, and tracking calories burned. These features can make it easier for individuals to stick to a fitness regimen, leading to improved physical health. Additionally, fitness apps can provide social support and accountability, which can be motivating and help individuals stay on track with their fitness goals. Fitness apps can also offer personalized recommendations and advice based on an individual’s fitness level, goals, and preferences, which can help individuals choose the most effective and enjoyable forms of physical activity for their needs. Fitness apps positively affect physical health, highlighting the potential for fitness applications to provide a convenient and effective way to improve physical health and promote healthy lifestyle habits (10). Fitness apps positively affect psychological health “suggests that using fitness applications on smartphones and other mobile devices can positively impact a person’s mental well-being (24–26). Physical activity has been well-documented to affect mental health positively, and fitness apps can provide an easy and convenient way for individuals to incorporate regular physical activity into their lives. By tracking progress, setting goals, and monitoring the impact of physical activity on mental well-being, fitness apps can help individuals see the direct benefits of physical activity for their mental health. Furthermore, fitness apps can also provide social support and accountability, which can be motivating and help individuals stay on track with their fitness goals. It can increase self-esteem and a sense of accomplishment, positively impacting mental health. In addition, fitness apps can also provide personalized recommendations and advice based on an individual’s fitness level, goals, and preferences, which can help individuals choose the most effective and enjoyable forms of physical activity for their needs. It can lead to increased engagement in physical activity and a more positive relationship with exercise, which can improve mental health. So, we proposed the following hypotheses;

*H6*: Fitness apps have positive effects on physical health.

*H7*: Fitness apps have positive effects on psychological health.

Fitness apps positively influence virtual reality exercise, and fitness applications can enhance and complement the experience of virtual reality exercise, leading to improved physical and mental health outcomes (15). Fitness apps can provide a range of features, including workout routines, tracking progress, setting goals, and monitoring the impact of physical activity on overall health (8). By integrating with virtual reality exercise, these features can make the virtual reality workout experience more personalized, engaging, and effective. For example, fitness apps can provide customized recommendations for virtual reality exercises based on an individual’s fitness level, goals, and preferences, which can help individuals choose the most effective and enjoyable forms of physical activity. In addition, fitness apps can provide real-time feedback and progress tracking during virtual reality exercises, which can help individuals monitor their progress and stay motivated. Fitness apps can also provide social support and accountability, enhancing the virtual reality workout experience and assisting individuals in staying on track with their fitness goals (24–26). It can increase engagement in virtual reality exercise and improve physical and mental health outcomes. Fitness apps positively impact the relationship between COVID-19 preventive strategies and virtual reality exercise (VRE). Given the ongoing COVID-19 pandemic, many individuals seek new and innovative ways to stay active and maintain their physical health. One such method is virtual reality exercise (VRE), which uses virtual reality technology to simulate physical activity. So we proposed the following hypotheses;

*H8*: Fitness apps have a positive influence on virtual reality exercise (VRE).

*H9*: Fitness apps positively moderate the relationships from COVID-19 preventive strategies to virtual reality exercise (VRE).

Fitness apps positively impact the relationship between virtual reality exercise (VRE) and psychological health. In other words, it posits that using fitness apps can help facilitate positive effects. Virtual reality exercise (VRE) involves using virtual reality technology to simulate physical activity and has become increasingly popular as a way to stay active and healthy during the COVID-19 pandemic. The hypothesis suggests that fitness apps can enhance the relationship between VRE and psychological health by providing users with personalized workout plans, tracking their progress, and offering motivation and support. By making VRE more accessible and convenient through fitness apps, individuals may be more likely to engage in regular physical activity, which could improve psychological well-being (24–26). In short, fitness apps can help foster the positive relationship between virtual reality exercise (VRE) and psychological health by providing individuals with the tools and support they need to engage in regular VRE, which could lead to improved mental health. In other words, it posits that using fitness apps can help facilitate the positive effects of virtual reality exercise (VRE) on an individual’s physical health. Virtual reality exercise (VRE) involves using virtual reality technology to simulate physical activity and has become increasingly popular as a way to stay active and healthy during the COVID-19 pandemic. Fitness apps can help foster the positive relationship between virtual reality exercise (VRE) and physical health by providing individuals with the tools and support they need to engage in regular VRE, which could lead to improved health outcomes. So we proposed the following hypotheses:

*H10*: Fitness apps positively moderate the relationships from virtual reality exercise (VRE) to psychological health.

*H11*: Fitness apps positively moderate the relationships from virtual reality exercise (VRE) to physical health.

**Figure 1:**
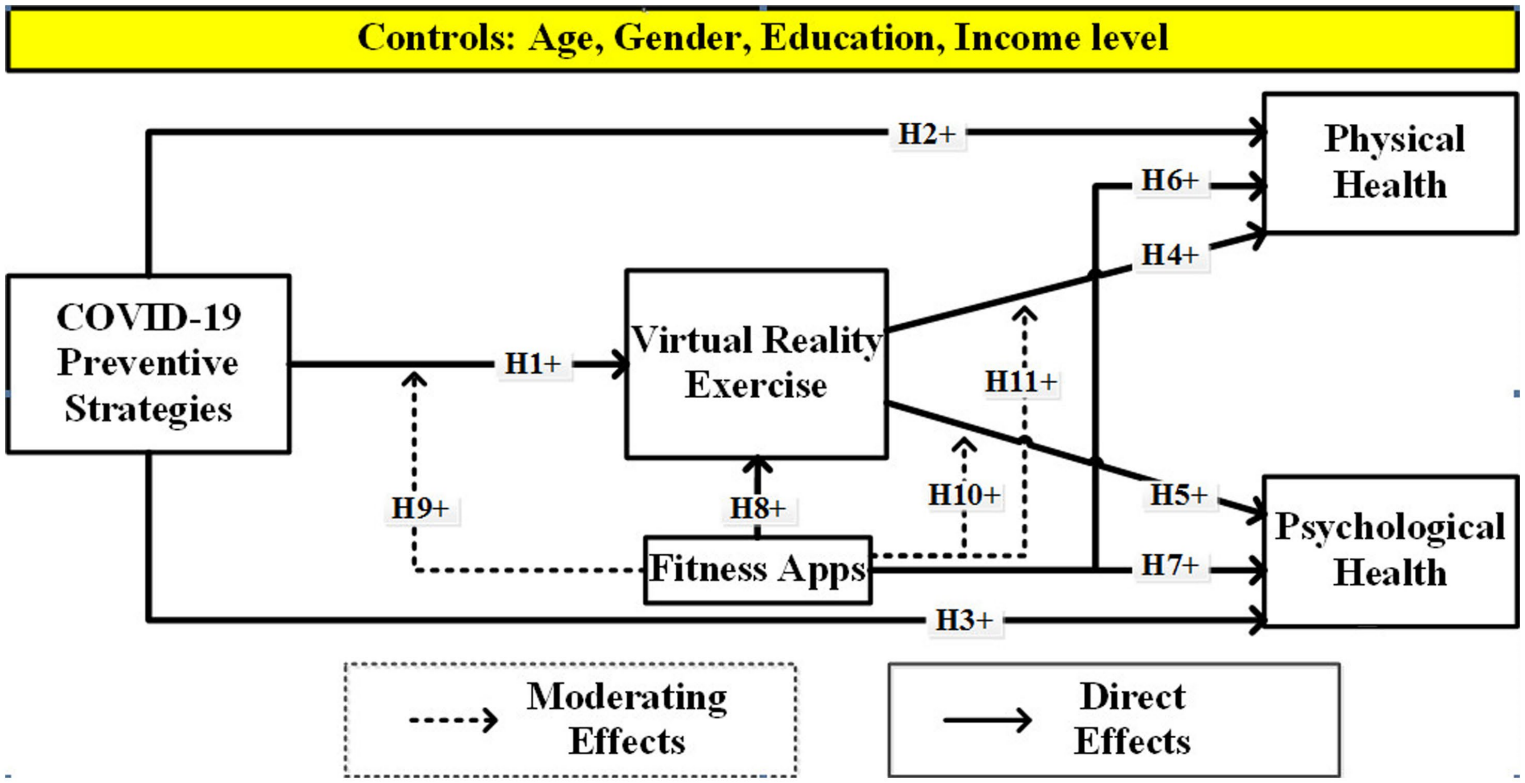
Proposed hypothetical model.

To conclude, we designed the hypothetical model according to the study objectives (see Figure 1).

## Methodology

### Study design

An online questionnaire survey was conducted under a cross-sectional design to explore how fitness apps moderate the links among COVID-19 preventive strategies, virtual reality exercise, physical health, and psychological health in Chinese society. A web-based platform (wjx.cn) was used for data collection under non-probability sampling (snowball). The research adhered to the World Medical Helsinki Policy requirements. The studies involving human participants were reviewed and approved by the ethics committee of Soochow University.

### Participants recruitment

Participants were recruited under defined inclusion and exclusion criteria. The inclusion criteria were individuals must use a digital platform (fitness apps, virtual reality exercise for physical and psychological health) during the COVID-19 preventive strategies promulgation (27). The exclusion criteria were those individuals who cannot participate in the current research and do not use digital platforms during the COVID-19 preventive strategies promulgation under the WHO digital health guideline. A total of 3,000 participants were recruited for the current study across China (see Supplementary File S1).

### Data collection tool

A deductive (top-down) methodology and rational approach were used to create a multi-item questionnaire. The top-down method examines the literature to locate existing items and create new items based on previously-established theories (28–30). The rational approach was used as a standardized procedure after developing the items/constructs to refine the multi-item questionnaire. The rational method for questionnaire development is based upon eight steps; theoretical framework, concept analysis, item specification, item production, item judgment, scale construction, validation, and comment. A team of six public health professionals examined the preliminary multi-item questionnaire under the rational method. Two independent translators translated (forward and backward) the questionnaire from its original language to the targeted one (31).

### Survey measures

#### COVID-19 preventive strategies

Most countries across the globe have implemented travel bans to cut off the COVID-19 virus transmission. Isolation at home for extended periods and travel restrictions between and within nations are examples of COVID-19 preventive strategies. A significant fitness and health problem for the afflicted population is forced physical inactivity (32). We asked Likert scale (1–5) based questions (stay at home, social distancing, wearing a face mask, home quarantine, active lifestyle, and maintaining personal hygiene) from our participants related to their behavior toward the preventive strategies. The World Health Organization (WHO) announced several actions to stop the spread of COVID-19 for public health and safety (32).

#### Virtual reality exercise

Aside from the gaming business, individual households have recently begun using virtual and augmented reality technology. With augmented reality (AR), virtual features are added to the physical environment and superimposed to provide real-time information or foster immersion (33). We asked Likert scale (1–5) based questions (VRE helpful for active living, VRE helpful for health wellness, VRE beneficial for achieving the required physical activity level) from our participants related to the virtual reality-based exercise. Virtual reality training integrates virtual reality capabilities with standard exercise machines like treadmills and bikes. Mixed with exercise equipment called virtual reality fitness, virtual reality benefits psychological and physical health (34).

#### Physical and psychological health

COVID-19 influences people’s psychological and physical health as well as their level of loneliness. The scourge of loneliness is prompting the development of more technology and social tools (35). Lack of physical activity impacts the body’s natural physiological functions, which may lead to muscular atrophy, an unstable energy balance, and decreased exercise capacity (36). We defined Likert scale (1–5) based constructs (how using fitness apps and virtual reality exercise impacted physical health positively) related to physical health during the COVID-19 pandemic. Technology is being used to improve mental wellness in addition to physical health. For instance, it has been shown that cognitive-behavioral techniques may considerably enhance health monitoring and prognosis when dealing with chronic disease and the difficulties that go along with it (37).

#### Fitness apps

Increased screen time has been shown to affect people’s health, happiness, sleep habits, and overall quality of life. Research shows that social isolation similarly affects people’s levels of physical activity. Therefore, individuals may maintain their fitness and health by engaging in home-based activities made possible by smartphone fitness programs (38). We asked Likert scale (1–5) based questions related to the fitness apps based physical activity and how fitness apps moderate the links among COVID-19 preventive strategies, virtual reality exercise, physical health, and psychological health. People may track their exercise progress, get feedback on their technique, and learn how to improve their workouts with the help of technology (26, 39).

#### Data collection

A nationwide online survey across China was conducted for data collection between February and June 2022 under non-probability sampling. Informed consent was taken from all research participants for using their responses for research purposes. The respondents were informed of the survey’s purpose and given 14 days to understand and submit it. Before the final poll, a pre-test with 55 respondents was undertaken to estimate the reliability and validity. A total of 3,000 questionnaires were distributed across China via online platforms. A total of 2,795 complete detailed replies were included in the final analysis.

#### Data analysis

Structural equation modeling techniques were employed to analyze the collected data through Smart-PLS 3.0 statistical tools (40). Data analysis under SEM includes two steps. First, use the measurement model to assess concept, reliability, and convergent validity. Second, mediation analysis created a structural equation model to test the study hypotheses. Mediation analysis uses a mediator variable to divide the exposure-outcome relationship into direct and indirect effects. Researchers typically employ the smart-partial least squares structural equation modeling (PLS-SEM) approach to develop reasoning in exploratory research. Practical benefits of Smart-PLS-SEM include several multiple regression models, factor analysis, path coefficients analysis, confirmatory factor prediction equation, and covariance structural models (41).

## Results

### Descriptive statistics

Table 1 depicts the values of mean scores, standard deviation, excess kurtosis, and skewness values. It has been proven that all of the scales employed in this inquiry to determine the mean scores, standard deviation, excess kurtosis, and skewness values were consistently “reliable” and produced satisfactory results (see Table 1).

**Table 1:** Mean, standard deviation, kurtosis, and skewness of the study constructs.

Items	*M*	SD	EK	S_k_P
CPS1	3.813	1.066	0.917	−1.059
CPS2	3.807	1.048	0.955	−1.059
CPS3	3.828	1.075	0.813	−1.045
CPS4	3.814	1.076	0.920	−1.086
FA1	4.024	0.747	2.380	−1.022
FA2	3.949	0.798	0.971	−0.783
FA3	4.031	0.786	1.570	−0.962
FA4	3.960	0.826	1.641	−0.992
PH1	3.861	1.000	1.089	−1.014
PH2	3.917	0.985	1.386	−1.127
PH3	3.911	0.972	1.009	−0.981
PH4	3.904	0.963	1.308	−1.061
PsH1	3.886	1.004	1.205	−1.067
PsH2	3.912	0.976	1.568	−1.164
PsH3	3.939	0.966	1.228	−1.059
PsH4	3.879	0.980	1.257	−1.039
VRE1	3.932	0.962	1.647	−1.160
VRE2	3.928	0.961	1.314	−1.057
VRE3	3.920	0.949	1.363	−1.037
VRE4	3.930	0.927	1.614	−1.084

M, Mean; SD, Standard Deviation; EK, Excess Kurtosis; SkP, Skewness Karl Pearson’s; CSP, COVID-19 Preventive Strategies; FA, Fitness Apps; PH, Physical Health; PsH, Psychological Health; VRE, Virtual Reality Exercise.

### Physical activity and fitness apps statistics

**Figure 2:**
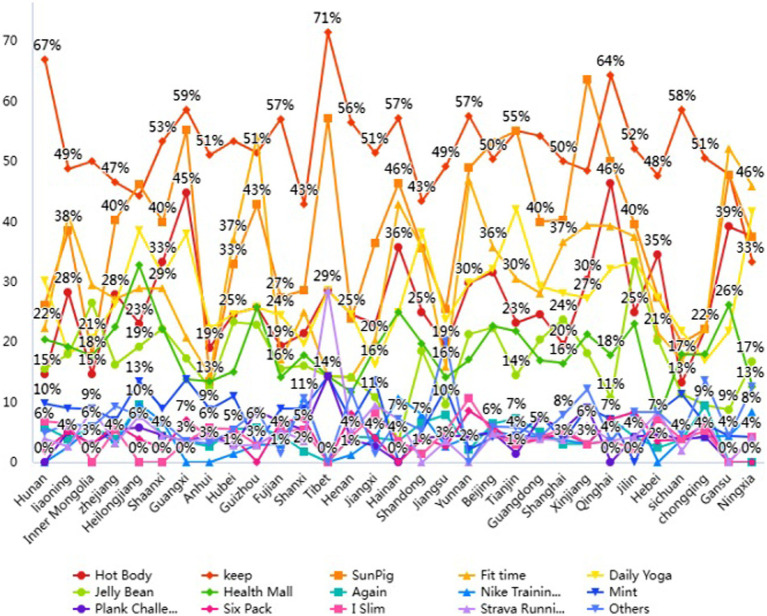
Physical activity and fitness apps statistics.

Figure 2 shows the distribution of the study participants’ physical activity and fitness apps during the COVID-19 pandemic. It has been found that KEEP was the favorite app among study participants used for physical activity and fitness. In addition, sun pig, hot body, daily yoga, fit time, health mall, jelly bean, and mint health are also getting popular across China.

### Virtual platforms for VR exercise

**Figure 3:**
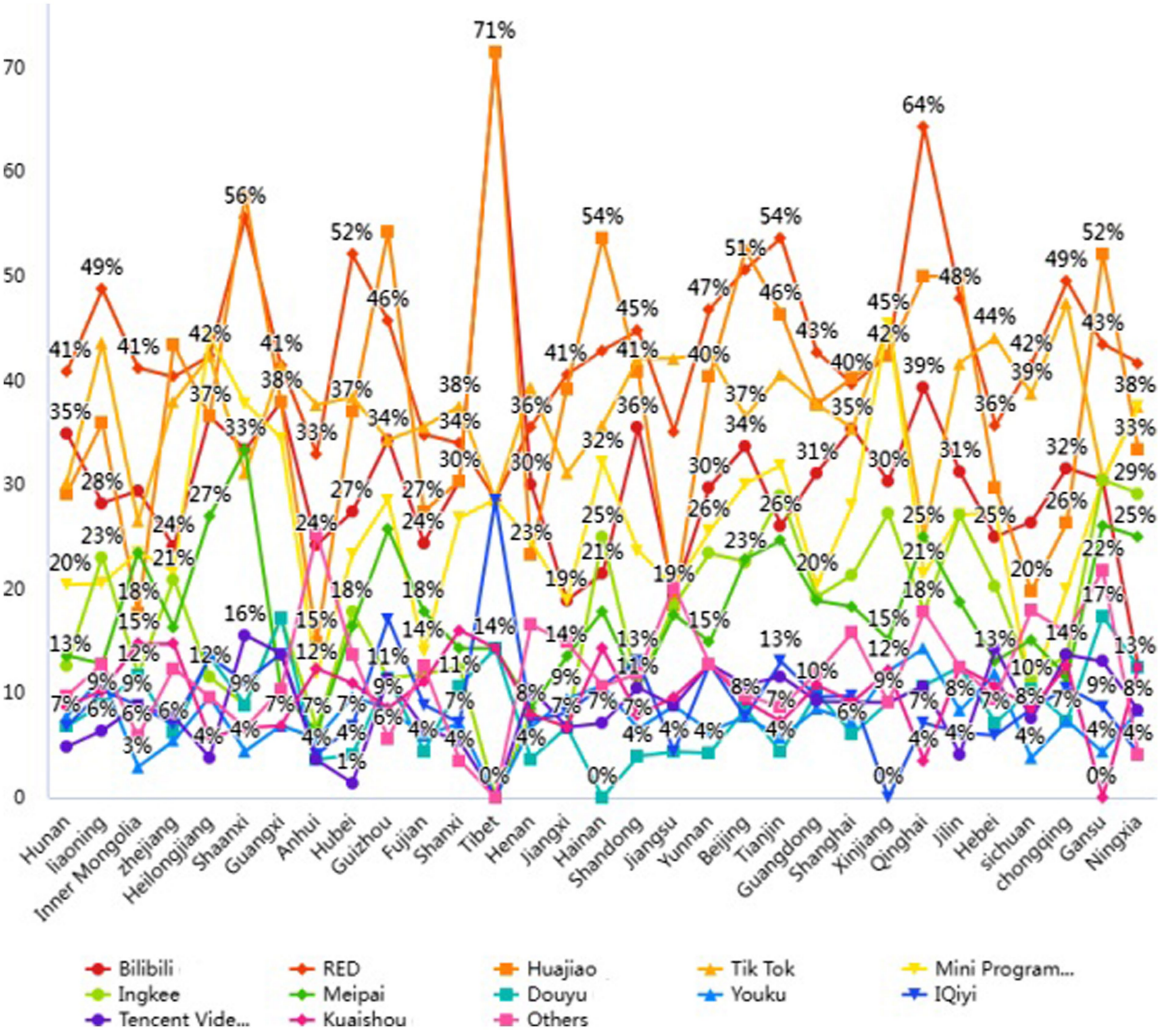
Virtual platforms for VR exercise.

Figure 3 shows the virtual platforms for VR-based exercise during the COVID-19 pandemic. According to the figure, famous platforms are bilibili, red, huajiao, tik tok, the mini program in wechat, ingkee, meipai, douyu, youku, iqiyi, tencent video, kuaishou, and others.

### Model measurement

In our study, factor analysis followed the construct reliability, discriminant validity, convergent validity, and content validity criteria. The reflective measurement model’s psychometric properties were also evaluated. The Cronbach’s alpha value for each construct was calculated to determine the reliability of the CPS, VRE, PH, PsH, and FA constructs. Computed Cronbach’s alpha values are more significant than the recommended limit of 0.70 (40). An estimate of the average variance collected was then used to assess the convergent validity (AVE). The test indicated that AVE values were above the limit of 0.50. We examined the AVEs, the Fornell-Larcker Criterion, construct reliability (CR), and Heterotrait-Monotrait (HTMT) correlation ratios for discriminant validity. We ensured that the indicator associations (square root of AVEs) with the assigned variables were higher than the correlation of all other factors (see Table 2). The variance inflation factor (VIF) was examined for all items of included constructs for multicollinearity. According to the results, the absence of multicollinearity is indicated by the estimated VIF values, which do not exceed 3 (see Table 3). VIFs suggest that the model might be evaluated structurally in more detail, and the requirements for a reflective measurement model might thus be satisfied. Further, the factor analysis confirmed that the proposed structural model is fit for hypothesis testing.

**Table 2:** Factor analysis.

Items	CPS	FA	PH	PsH	VRE	VIF	Alpha (α)	rho_A	CRs	AVEs
CPS1	**0.828**	0.257	0.238	0.212	0.370	1.947	0.868	0.868	0.910	0.716
CPS2	**0.869**	0.248	0.235	0.209	0.415	2.369				
CPS3	**0.873**	0.263	0.239	0.206	0.399	2.440				
CPS4	**0.814**	0.224	0.209	0.200	0.413	1.802				
FA1	0.214	**0.771**	0.199	0.206	0.262	1.626	0.837	0.841	0.891	0.671
FA2	0.243	**0.845**	0.230	0.219	0.331	1.966				
FA3	0.233	**0.834**	0.233	0.226	0.328	1.878				
FA4	0.268	**0.825**	0.230	0.251	0.284	1.846				
PH1	0.221	0.239	**0.826**	0.312	0.251	1.869	0.870	0.871	0.911	0.719
PH2	0.224	0.224	**0.852**	0.212	0.182	2.256				
PH3	0.242	0.225	**0.853**	0.214	0.250	2.145				
PH4	0.235	0.236	**0.861**	0.217	0.227	2.224				
PsH1	0.205	0.232	0.481	**0.828**	0.217	1.915	0.862	0.865	0.906	0.706
PsH2	0.191	0.219	0.237	**0.839**	0.202	2.091				
PsH3	0.197	0.222	0.221	**0.836**	0.228	2.011				
PsH4	0.224	0.249	0.302	**0.858**	0.254	2.066				
VRE1	0.378	0.300	0.222	0.215	**0.810**	1.860	0.815	0.816	0.878	0.644
VRE2	0.394	0.301	0.231	0.226	**0.811**	1.845				
VRE3	0.370	0.283	0.202	0.212	**0.789**	1.744				
VRE4	0.374	0.301	0.210	0.211	**0.798**	1.770				

CSP, COVID-19 Preventive Strategies; FA, Fitness Apps; PH, Physical Health; PsH, Psychological Health; VRE, Virtual Reality Exercise; CRs, Critical Ratios; AVE, Average Variance Extracted.

**Table 3:** Discriminant validity.

Constructs	Heterotrait-Monotrait Ratio (HTMT)					Fornell-Larcker Criterion				
	CPS	FA	PH	PsH	VRE	CPS	FA	PH	PsH	VRE
CPS	1					0.846				
FA	0.343	1				0.293	0.819			
PH	0.313	0.319	1			0.272	0.273	0.848		
PsH	0.281	0.323	0.701	1		0.244	0.275	0.606	0.840	
VRE	0.561	0.445	0.318	0.320	1	0.472	0.369	0.270	0.269	0.802

CSP, COVID-19 Preventive Strategies; FA, Fitness Apps; PH, Physical Health; PsH, Psychological Health; VRE, Virtual Reality Exercise.

### PLS-SEM statistics

The SEM technique often allows the calculation and testing of many relationships simultaneously in a single projected model with many links instead of examining each connection individually. The smart-PLS-SEM method was used in this study to analyze the suggested structural model. To use the SEM, we first determine the structural model’s fitness (see Table 4). The standardized residuals between the hypothesized and observed covariance matrices are standardized, and the average index of these residuals is known as the SRMR value. It also evaluates how well a forecasted and suggested model fits with the gathered data (42). To indicate that the presented model is a good fit and acceptable, the SRMR value must be identical and be less than 0.08. The results match the model well (see Table 4).

**Table 4:** Model fitness.

Fit indices	Saturated model
SRMR	0.044
d_ULS	0.414
d_G	0.168
Chi-Square	2,862.521
NFI	0.895

SRMR, Standardized-root-mean-square-residual; d_ULS, Unweighted least squares discrepancy; d_G, Geodesic discrepancy; X2, Chi-square; NFI, Normed fit index.

**Figure 4:**
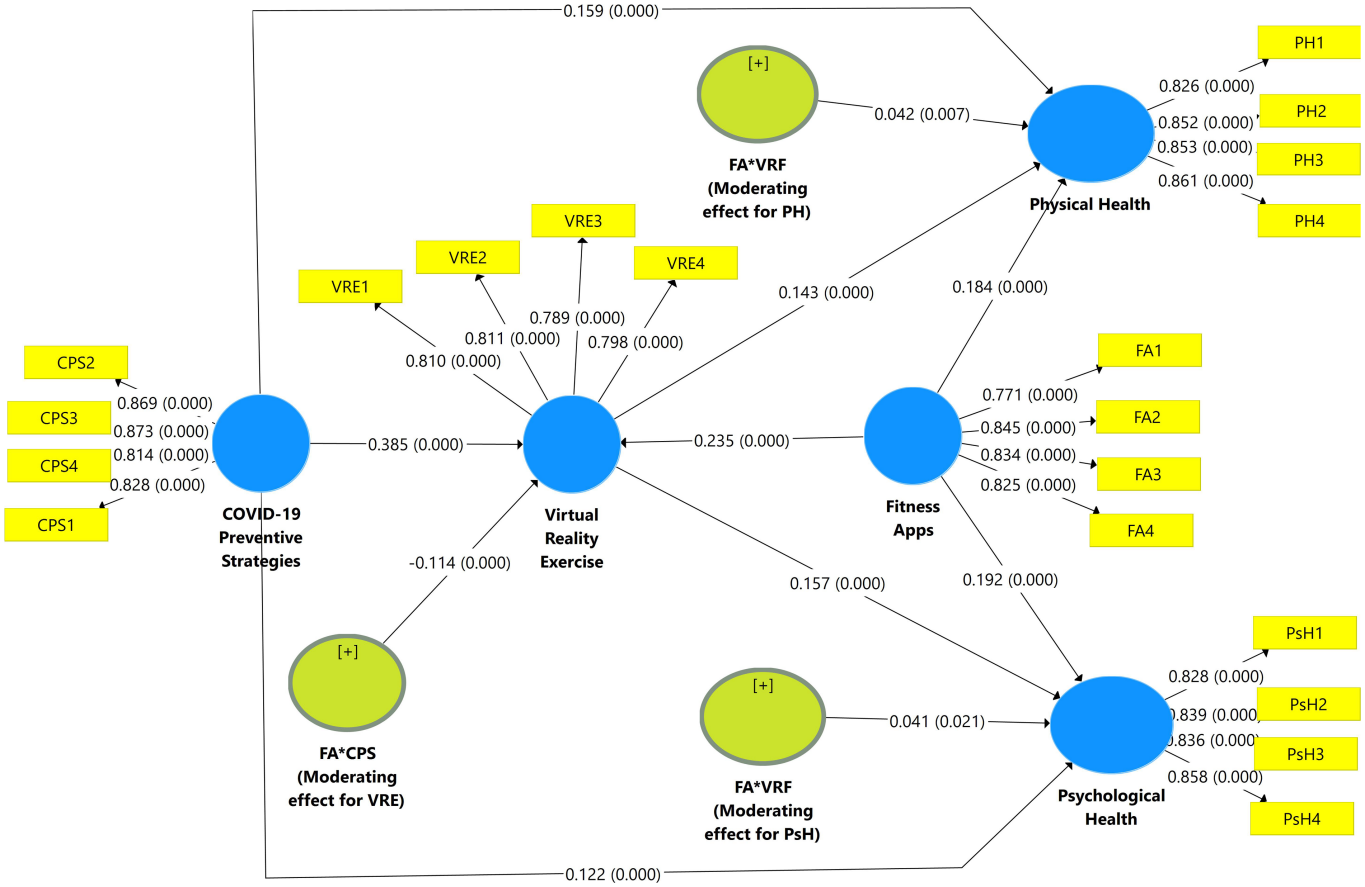
PLS-SEM results.

For path coefficients and *t*-value estimation, we calculate the standardized beta (*β*) value to assess the importance of the presented hypothesis. When testing for unit-variation in the independent-variable (COVID-19 Preventive Strategies) constructions, the beta (*β*) value describes the most likely range of dependent-variable constructs to observe the direct and moderating effects of COVID-19 Preventive Strategies and fitness apps toward physical and psychological health. So, for the proposed model, we determined a beta (*β*) value for each possible course of action. Higher and significant beta (*β*) levels will have a more extensive influence on endogenous-latent constructs. The *t*-test is a method for assessing the level of significance of the beta (*β*) value. We employed bootstrapping SEM technique to evaluate the significance of the presented hypothesis (see Table 5; Figure 4).

**Table 5:** Smart PLS-SEM results.

Hypothetical relationships	Beta (*β*)	Standard Deviation	T Statistics	*P* Values
H1 = CPS -> VRE	0.385	0.025	15.699	0.000
H2 = CPS -> PH	0.159	0.021	7.405	0.000
H3 = CPS -> PsH	0.122	0.022	5.435	0.000
H4 = VRE -> PH	0.143	0.022	6.493	0.000
H5 = VRE -> PsH	0.157	0.024	6.444	0.000
H6 = FA -> PH	0.184	0.020	9.071	0.000
H7 = FA -> PsH	0.192	0.021	9.319	0.000
H8 = FA -> VRE	0.235	0.020	11.889	0.000
H9 = FA*CPS (Moderating effect for VRE) -> VRE	−0.114	0.023	4.872	0.000
H10 = FA*VRF (Moderating effect for PH) -> PH	0.042	0.015	2.872	0.004
H11 = FA*VRF (Moderating effect for PsH) -> PsH	0.041	0.015	2.699	0.007

CSP, COVID-19 Preventive Strategies; FA, Fitness Apps; PH, Physical Health; PsH, Psychological Health; VRE, Virtual Reality Exercise.

The results of H1 (*β* = 0.385, *t* = 15.699, *p* = 0.000) showed statistically significant at the 5% level for COVID-19 preventive methods’ positive effects on virtual reality exercise. Consequently, the findings confirm H1, which asserted that CPS had a favorable impact on VRE. Hypothesis 2 stated, “COVID-19 preventive strategies positively affect physical health.” In Table 5, results endorse a positive effect of CPS toward PH with (*β* = 0.159, *t* = 7.405, *p* = 0.000), which supports H2. H3 results (*β* = 0.122, *t* = 5.435, *p* = 0.000) show that CPS positively influences psychological health, which is significantly supported. In a hypothetical model (see Figure 1), H4 and H5 were proposed to examine the direct effect of VRE on PH (H4) and PsH (H5). The outcomes show that VRE has a significant and positive effect on PH (*β* = 0.143, *t* = 6.493, *p* = 0.000) and PsH (*β* = 0.157, *t* = 6.444, *p* = 0.000), respectively supported. In the research framework, three hypotheses were proposed for the examination of fitness apps’ influence on PH (H6), PsH (H7), and VRE (H8). Results for the direct effect of the FA concept endorsed that there are positive and significant effects on PH (*β* = 0.184, *t* = 9.071, *p* = 0.000), PsH (*β* = 0.192, *t* = 9.319, *p* = 0.000), and VRE (*β* = 0.235, *t* = 11.899, *p* = 0.000). The overall results explained that using fitness apps is beneficial and supported.

Moreover, we employed the product-indicator approach in moderation analysis. According to the results, hypotheses 9, 10, and 11 stated,” fitness apps positively moderate the relationships from CPS to VRE (H9), VRE to PsH (H10), and VRE to PH (H11).” Overall results for moderating effects of fitness apps motivated the relationships among CPS, VRE, PH, and PsH constructs. Thus, the final results of Table 5 endorse the significant moderating effect of FA on the relationship among CPS and VRE with (*β* = −0.114, *t* = 4.872, *p* = 0.000), which shows as unfavorable, which was not supported to proposed hypothesis H9. The moderating effect of FA on relationships among VRE and PH (*β* = 0.042, *t* = 2.872, *p* = 0.004) as well as VRE and PsH (*β* = 0.041, *t* = 2.699, *p* = 0.007), which were supported to the proposed hypothesis in the model (see Figure 1; Table 5).

## Discussion

Most countries have instituted travel bans to stem the tide of the SARS-CoV-2 infection. Millions of people have been compelled to adopt an abnormally inactive lifestyle due to the unexpected closure of gyms, sports grounds, and parks and firm limits to outdoor movement. Concerns about the fitness and well-being of the afflicted population are warranted due to the combination of forced physical inactivity and the emergence of undesirable coping methods, including smoking, drinking, and food cravings (43). Sports and healthy lifestyles are only two areas where the widespread use of digital technology had a substantial positive influence. Wearable and fitness apps do these functions, including monitoring physical activity, comparing user data, and establishing online communities (44). Our study results also align with the above-reported findings, and (43, 44) show that Chinese people are using more wearable and fitness apps for digital sports and physical exercise to maintain a healthy lifestyle. Regular gym-goers and members were looking for options, and many found what they were looking for in the form of customized, at-home, and probably digital sporting activities. Experts in the field of public health propose using digital tools for at-home exercises during the pandemic (45). Regular exercise has been shown to serve crucial functions in human existence. Frequent and regular exercise helps maintain a healthy weight, reduces feelings of despair and anxiety, and decreases the risk of developing disorders like mental decline (46).

The market for fitness apps designed specifically for smartphones has expanded rapidly in recent years. Smartphone applications provide extensive capabilities and advantages in mental health care (47). By leveraging the capabilities and widespread availability of mobile phones worldwide, mHealth (or mobile health) is a new approach to healthcare that improves two-way communication between patients and healthcare benefactors, expands contact with health services, and encourages a healthy lifestyle (48). An international investigation by the World Health Organization (WHO) demonstrates several applications of mobile devices in healthcare communication. Technology like this is being used to better; emergency-service provider-provider communication, patient-health monitoring, surveillance, and the healthcare system as a whole (49). Our study findings show that fitness apps and virtual reality exercise positively promote physical and psychological health in COVID-19 preventive strategies because of convenience and accessibility. Similar results were reported by (24, 50) that extensive reach, accessibility, and comfort, physical activity mobile applications provide a novel way to encourage participation in physical exercise. Commercial physical activity applications are now more widely available than ever before. Four different digital technologies are among the top 20 fitness trends according to the ACSM’s Health & Fitness Journal’s Worldwide Survey of Fitness Trends 2021. Due to self-isolation and quarantine regulations, fewer chances to be physically active, and fear of contracting COVID-19, the atmosphere produced by the pandemic encourages lower levels of habitual physical activity. Sedentary lifestyles and prolonged physical inactivity are often linked to ill physical and mental health and an elevated risk of disease-specific and all-cause death (51). Mobile health (mHealth), which uses cell phones for health-related apps, has become an essential tool for interventions to change behavior for the better and prevent health issues (52).

After two years at the top, wearable technology is now placed second. Fitness applications are ranked 12th, and virtual training ranks sixth (25, 53). KEEP, for instance, has more than 30 million users, making it one of China’s most widely used fitness applications (54). Most of our study participants also used KEEP fitness apps for an active lifestyle. Mobile apps are a potential tool for promoting at-home adherence to physical activity. Information and communication technology is frequently used in educational practices and physical health. Technology-based treatments encouraging physical activity are a good substitute for conventional medical care (33). Fitness treatment in virtual reality has a profoundly favorable effect on wellness. Virtual reality exercises for treating chronic disorders get minimal attention, and virtual reality-based treatments’ influence on anxiety mainly focuses on virtual reality training. Numerous psychological and physical advantages are linked to regular physical exercise. The cognition hypothesis outlined how people’s perceptions of events affect their emotional and behavioral responses. Both physical and psychological health has been shown to benefit from exercise. Regular physical exercise is crucial for easing isolation’s effects on mental health and maintaining a robust immune system (55). Virtual reality mixed with exercise equipment, called virtual reality fitness, increases the psychological advantages of exercise and the likelihood that individuals would exercise regularly. Our study findings and (33, 54) show that using fitness apps and virtual reality exercise positively influences physical and psychological health. Similarly, there is a rise in the use of virtual cycling in March, particularly in nations like Italy and Spain, for physical and psychological health (56, 57).

## Conclusion

Users use fitness apps to plan their workouts, keep their eyes on their progress, get new ideas, and brag about their achievements on social media. Ease of use, automated monitoring, and safety are the features of fitness apps and virtual reality exercises that promote health protection motivation. Individuals experimented with a wide range of home-based workout strategies. The fitness app’s primary purpose during and after the pandemic is to motivate users to keep up with their regular at-home workouts and to utilize physical activity to keep their minds healthy. Seventy percent of deaths worldwide are attributable to non-communicable diseases, and exercise may help with prevention and therapy using fitness and healthcare apps for an active and healthy lifestyle. The social cognitive tradition has long been the dominant paradigm in the study of physical activity and has shed light on many significant themes related to physical exertion. Research on the humanistic framework for understanding physical exercise has increased dramatically over the last decade, and its results have shown early promise for explaining and altering behavior.

### Implications

The relationship between new technologies and sedentary behavior is nuanced and multifaceted. Some new technology (such as video and computer games) is to blame for the widespread problem of inactivity that has developed in recent decades. However, other cutting-edge technologies have been widely used to advocate for exercise and health. Healthcare professionals may be crucial in evaluating and adopting new technologies. The information in this area will provide readers with a solid grounding in applying cutting-edge technology in assessing and promoting physical activity. We anticipate this information will further encourage researchers and medical experts to use cutting-edge physical activity and health technology. Fitness technology is effective in getting sedentary people to participate in physical exercise.

### Limitations

The current study has several limitations (snowball sampling, cross-sectional research design, dependent on fitness apps and virtual platforms, self-administered questionnaire, rational and deductive approach for questionnaire construction). Snowball sampling is a non-probability sampling technique where participants are recruited through referrals from existing participants. Due to the COVID-19 restrictions, we recruited study participants using the snowball sampling technique because it is a helpful method for accessing the hard-to-reach population. A cross-sectional research design was used in the study, meaning that data is collected from different individuals at a single point in time. This design helps examine relationships between variables at a specific moment. The study’s limitations stem from the fact that the participants must use fitness apps and virtual platforms for their psychological and physical health. This criterion introduces a selection bias, as individuals who do not use such technologies or prefer alternative health maintenance methods are excluded. The self-administered questionnaire was developed using a rational and deductive approach to questionnaire construction. While this approach allows for a systematic development process based on theoretical frameworks and existing literature, it may limit the inclusion of novel or unexpected constructs that could emerge from a more exploratory approach.

## Data availability statement

The original contributions presented in the study are included in the article/Supplementary material, further inquiries can be directed to the corresponding author.

## Ethics statement

The studies involving human participants were reviewed and approved by Ethics committee of Soochow University. The patients/participants provided their written informed consent to participate in this study.

## Author contributions

RM is the principal investigator and overall wrote and edited the manuscript. MY contributed to drafting the literature review and discussion. LQ and ZS performed the statistical analysis. All authors contributed to the article and approved the submitted version.

## Funding

This study was supported by a grant from Department of Education of Guangdong Province (No. 2022KCXTD027) and Guangdong Key Construction Discipline Research Ability Enhancement Project (2021ZDJS108).

## Conflict of interest

The authors declare that the research was conducted in the absence of any commercial or financial relationships that could be construed as a potential conflict of interest.

## Publisher’s note

All claims expressed in this article are solely those of the authors and do not necessarily represent those of their affiliated organizations, or those of the publisher, the editors and the reviewers. Any product that may be evaluated in this article, or claim that may be made by its manufacturer, is not guaranteed or endorsed by the publisher.
