# Supplementary material for: The association between COVID-19 preventive strategies, virtual reality exercise, use of fitness apps, physical, and psychological health: testing a structural equation moderation model
Source: Front Public Health. 2023 Jul 6;11:1170645. doi: 10.3389/fpubh.2023.1170645 (PMC10358774; doi:10.3389/fpubh.2023.1170645)
Supplement: Supplementary file 1 [file Table_1.DOCX]

**Population Characteristics**

X= Demographics Y= Region

| X/Y | **Gender** | | **Age Groups (Years)** | | | | | | **Education** | | | | | | **Marital Status** | | | | |
| --- | --- | --- | --- | --- | --- | --- | --- | --- | --- | --- | --- | --- | --- | --- | --- | --- | --- | --- | --- |
|  | Male | Female | 21-22 | 23-29 | 30-36 | 37-42 | 43-49 | 50+ | T&V  College level | Bachelor | Associate  Degree | Master | Graduate | Others | Married | | Un-  married | | Others |
| Hunan | 57(55.34%) | 46(44.66%) | 11(10.68%) | 43(41.75%) | 23(22.33%) | 13(12.62%) | 10(9.71%) | 3(2.91%) | 4(3.88%) | 4(3.88%) | 21(20.39%) | 30(29.13%) | 35(33.98%) | 9(8.74%) | 68(66.02%) | | 35(33.98%) | | 0(0.00%) |
| Liaoning | 30(38.46%) | 48(61.54%) | 6(7.69%) | 27(34.62%) | 20(25.64%) | 12(15.38%) | 7(8.97%) | 6(7.69%) | 3(3.85%) | 5(6.41%) | 18(23.08%) | 38(48.72%) | 9(11.54%) | 5(6.41%) | 62(79.49%) | | 15(19.23%) | | 1(1.28%) |
| Inner Mongolia | 13(38.24%) | 21(61.76%) | 3(8.82%) | 13(38.24%) | 12(35.29%) | 3(8.82%) | 2(5.88%) | 1(2.94%) | 2(5.88%) | 3(8.82%) | 12(35.29%) | 9(26.47%) | 8(23.53%) | 0(0.0%) | 23(67.65%) | | 9(26.47%) | | 2(5.88%) |
| Zhejiang | 78(60.47%) | 51(39.53%) | 14(10.85%) | 45(34.88%) | 35(27.13%) | 13(10.08%) | 12(9.30%) | 10(7.75) | 9(6.98%) | 10(7.75%) | 39(30.23%) | 42(32.56%) | 25(19.38%) | 4(3.10%) | 100(77.52%) | | 27(20.93%) | | 2(1.55%) |
| Heilongjiang | 34(65.38%) | 18(34.62%) | 5(9.62%) | 14(26.92%) | 11(21.15%) | 11(21.15%) | 6(11.54%) | 5(9.62%) | 2(3.85%) | 4(7.69%) | 15(28.85%) | 25(48.08%) | 6(11.54%) | 0(0.0%) | 43(82.69%) | | 9(17.31%) | | 0(0.00%) |
| Shaanxi | 26(57.78%) | 19(42.22%) | 7(15.56%) | 19(42.22%) | 5(11.11%) | 6(13.33%) | 6(13.33%) | 2(4.44%) | 1(2.22%) | 5(11.11%) | 18(40%) | 16(35.56%) | 5(11.11%) | 0(0.0%) | 37(82.22%) | | 8(17.78%) | | 0(0.00%) |
| Guangxi | 15(51.72%) | 14(48.28%) | 1(3.45%) | 14(48.28%) | 6(20.69%) | 3(10.34%) | 3(10.34%) | 2(6.90%) | 1(3.45%) | 1(3.45%) | 8(27.59%) | 13(44.83%) | 5(17.24%) | 1(3.45%) | 24(82.76%) | | 3(10.34%) | | 2(6.90%) |
| Anhui | 93(47.94%) | 101(52.06%) | 24(12.37%) | 83(42.78%) | 57(29.38%) | 11(5.67%) | 16(8.25%) | 3(1.55%) | 15(7.73%) | 22(11.34%) | 49(25.26%) | 33(17.01%) | 61(31.44%) | 14(7.22%) | 115(59.28%) | | 70(36.08%) | | 9(4.64%) |
| Hubei | 40(54.79%) | 33(45.21%) | 9(12.33%) | 32(43.84%) | 15(20.55%) | 7(9.59%) | 7(9.59%) | 3(4.11%) | 4(5.48%) | 5(6.85%) | 19(26.03%) | 30(41.10%) | 12(16.44%) | 3(4.11%) | 54(73.97%) | | 19(26.03%) | | 0(0.00%) |
| Guizhou | 18(51.43%) | 17(48.57%) | 6(17.14%) | 11(31.43%) | 8(22.86%) | 5(14.29%) | 3(8.57%) | 2(5.71%) | 0(0.00%) | 2(5.71%) | 13(37.14%) | 14(40%) | 6(17.14%) | 0(0.00%) | 28(80%) | | 6(17.14%) | | 1(2.86%) |
| Fujian | 74(54.81%) | 61(45.19%) | 18(13.33%) | 43(31.85%) | 41(30.37%) | 16(11.85%) | 13(9.63%) | 4(2.96%) | 9(6.67%) | 19(14.07%) | 38(28.15%) | 34(25.19%) | 27(20%) | 8(5.93%) | 106(78.52%) | | 28(20.74%) | | 1(0.74%) |
| Shanxi | 29(51.79%) | 27(48.21%) | 2(3.57%) | 18(32.14%) | 21(37.5%) | 7(12.5%) | 6(10.71%) | 2(3.57%) | 2(3.57%) | 8(14.29%) | 20(35.71%) | 18(32.14%) | 5(8.93%) | 3(5.36%) | 45(80.36%) | | 10(17.86%) | | 1(1.79%) |
| Tibet | 3(42.86%) | 4(57.14%) | 1(14.29%) | 2(28.57%) | 1(14.29%) | 3(42.86%) | 0(0.00%) | 0(0.00%) | 0(0.00%) | 1(14.29%) | 3(42.86%) | 2(28.57%) | 1(14.29%) | 0(0.00%) | 5(71.43%) | | 2(28.57%) | | 0(0.00%) |
| Henan | 74(45.40%) | 89(54.60%) | 13(7.98%) | 75(46.01%) | 38(23.31%) | 18(11.04%) | 17(10.43%) | 2(1.23%) | 9(5.52%) | 28(17.18%) | 35(21.47%) | 32(19.63%) | 48(29.45%) | 11(6.75%) | 106(65.03%) | | 51(31.29%) | | 6(3.68%) |
| Jiangxi | 34(45.95%) | 40(54.05%) | 11(14.86%) | 25(33.78%) | 22(29.73%) | 7(9.46%) | 7(9.46%) | 2(2.70%) | 3(4.05%) | 4(5.41%) | 24(32.43%) | 16(21.62%) | 25(33.78%) | 2(2.70%) | 50(67.57%) | | 24(32.43%) | | 0(0.00%) |
| Hainan | 16(57.14%) | 12(42.86%) | 1(3.57%) | 9(32.14%) | 7(25%) | 2(7.14%) | 7(25%) | 2(7.14%) | 0(0.00%) | 4(14.29%) | 8(28.57%) | 11(39.29%) | 4(14.29%) | 1(3.57%) | 23(82.14%) | | 5(17.86%) | | 0(0.00%) |
| Shandong | 49(64.47%) | 27(35.53%) | 8(10.53%) | 30(39.47%) | 19(25%) | 8(10.53%) | 5(6.58%) | 6(7.89%) | 4(5.26%) | 10(13.16%) | 23(30.26%) | 25(32.89%) | 13(17.11%) | 1(1.32%) | 51(67.11%) | | 25(32.89%) | | 0(0.00%) |
| Jiangsu | 67(58.77%) | 47(41.23%) | 9(7.89%) | 32(28.07%) | 33(28.95%) | 21(18.42%) | 14(12.28%) | 5(4.39%) | 2(1.75%) | 7(6.14%) | 28(24.56%) | 30(26.32%) | 42(36.84%) | 5(4.39%) | 85(74.56%) | | 28(24.56%) | | 1(0.88%) |
| Yunnan | 18(38.30%) | 29(61.70%) | 2(4.26%) | 14(29.79%) | 10(21.28%) | 5(10.64%) | 8(17.02%) | 8(17.02%) | 0(0.00%) | 3(6.38%) | 11(23.40%) | 27(57.45%) | 5(10.64%) | 1(2.13%) | 39(82.98%) | | 6(12.77%) | | 2(4.26%) |
| Beijing | 205(52.70%) | 184(47.30%) | 31(7.97%) | 143(36.76%) | 66(16.97%) | 51(13.11%) | 66(16.97%) | 32(8.23%) | 12(3.08%) | 19(4.88%) | 119(30.59%) | 188(48.33%) | 48(12.34%) | 3(0.77%) | 329(84.58%) | | 55(14.14%) | | 5(1.29%) |
| Tianjin | 33(47.83%) | 36(52.17%) | 5(7.25%) | 27(39.13%) | 15(21.74%) | 8(11.59%) | 6(8.70%) | 8(11.59%) | 3(4.35%) | 12(17.39%) | 15(21.74%) | 28(40.58%) | 11(15.94%) | 0(0.00%) | 55(79.71%) | | 13(18.84%) | | 1(1.45%) |
| Guangdong | 134(51.54%) | 126(48.46%) | 31(11.92%) | 103(39.62%) | 56(21.54%) | 32(12.31%) | 30(11.54%) | 8(3.08%) | 8(3.08%) | 29(11.15%) | 72(27.69%) | 88(33.85%) | 56(21.54%) | 7(2.69%) | 199(76.54%) | | 58(22.31%) | | 3(1.15%) |
| Shanghai | 76(46.34%) | 88(53.66%) | 12(7.32%) | 58(35.37%) | 46(28.05%) | 18(10.98%) | 21(12.80%) | 9(5.49%) | 8(4.88%) | 11(6.71%) | 36(21.95%) | 56(34.15%) | 48(29.27%) | 5(3.05%) | 132(80.49%) | 31(18.90%) | | 1(0.61%) | |
| Xinjiang | 18(54.55%) | 15(45.45%) | 6(18.18%) | 11(33.33%) | 3(9.09%) | 3(9.09%) | 8(24.24%) | 2(6.06%) | 0(0.00%) | 4(12.12%) | 13(39.39%) | 12(36.36%) | 4(12.12%) | 0(0.00%) | 27(81.82%) | 6(18.18%) | | 0(0.00%) | |
| Qinghai | 16(57.14%) | 12(42.86%) | 2(7.14%) | 12(42.86%) | 3(10.71%) | 5(17.86%) | 5(17.86%) | 1(3.57%) | 0(0.00%) | 3(10.71%) | 7(25%) | 16(57.14%) | 2(7.14%) | 0(0.00%) | 24(85.71%) | 4(14.29%) | | 0(0.00%) | |
| Jilin | 25(52.08%) | 23(47.92%) | 5(10.42%) | 14(29.17%) | 14(29.17%) | 6(12.5%) | 8(16.67%) | 1(2.08%) | 2(4.17%) | 9(18.75%) | 17(35.42%) | 12(25%) | 7(14.58%) | 1(2.08%) | 41(85.42%) | 7(14.58%) | | 0(0.00%) | |
| Hebei | 48(57.14%) | 36(42.86%) | 3(3.57%) | 25(29.76%) | 20(23.81%) | 20(23.81%) | 12(14.29%) | 4(4.76%) | 1(1.19%) | 11(13.10%) | 28(33.33%) | 24(28.57%) | 19(22.62%) | 1(1.19%) | 75(89.29%) | 9(10.71%) | | 0(0.00%) | |
| Sichuan | 55(51.89%) | 51(48.11%) | 9(8.49%) | 45(42.45%) | 34(32.08%) | 8(7.55%) | 8(7.55%) | 2(1.89%) | 6(5.66%) | 9(8.49%) | 27(25.47%) | 29(27.36%) | 27(25.47%) | 8(7.55%) | 75(70.75%) | 28(26.42%) | | 3(2.83%) | |
| Chongqing | 50(52.63%) | 45(47.37%) | 30(31.58%) | 31(32.63%) | 21(22.11%) | 4(4.21%) | 7(7.37%) | 2(2.11%) | 3(3.16%) | 6(6.32%) | 17(17.89%) | 29(30.53%) | 39(41.05%) | 1(1.05%) | 54(56.84%) | 38(40%) | | 3(3.16%) | |
| Gansu | 10(43.48%) | 13(56.52%) | 2(8.70%) | 6(26.09%) | 6(26.09%) | 4(17.39%) | 3(13.04%) | 2(8.70%) | 1(4.35%) | 2(8.70%) | 8(34.78%) | 9(39.13%) | 3(13.04%) | 0(0.00%) | 20(86.96%) | 3(13.04%) | | 0(0.00%) | |
| Ningxia | 12(50%) | 12(50%) | 4(16.67%) | 9(37.5%) | 4(16.67%) | 3(12.5%) | 2(8.33%) | 2(8.33%) | 0(0.00%) | 2(8.33%) | 9(37.5%) | 10(41.67%) | 3(12.5%) | 0(0.00%) | 18(75%) | 6(25%) | | 0(0.00%) | |

**Duration of physical activity during the COVID-19 pandemic**

| **X\Y** | **Less than 30 minutes** | **30 to 60 minutes** | **60 minutes or more** | **Subtotal** |
| --- | --- | --- | --- | --- |
| Anhui | 53.61% | 28.35% | 18.04% | 194 |
| Beijing | 31.36% | 40.62% | 28.02% | 389 |
| Fujian | 43.70% | 37.78% | 18.52% | 135 |
| Gansu | 34.78% | 34.78% | 30.43% | 23 |
| Guangdong | 38.08% | 36.92% | 25% | 260 |
| Guangxi | 27.59% | 37.93% | 34.48% | 29 |
| Guizhou | 37.14% | 37.14% | 25.71% | 35 |
| Hainan | 28.57% | 42.86% | 28.57% | 28 |
| Hebei | 34.52% | 36.90% | 28.57% | 84 |
| Henan | 39.26% | 38.65% | 22.09% | 163 |
| Heilongjiang | 26.92% | 42.31% | 30.77% | 52 |
| Hubei | 30.14% | 35.62% | 34.25% | 73 |
| Hunan | 36.89% | 39.81% | 23.30% | 103 |
| Jilin | 25% | 50% | 25% | 48 |
| Jiangsu | 40.35% | 38.60% | 21.05% | 114 |
| Jiangxi | 47.30% | 28.38% | 24.32% | 74 |
| Liaoning | 44.87% | 30.77% | 24.36% | 78 |
| Inner Mongolia | 38.24% | 23.53% | 38.24% | 34 |
| Ningxia | 41.67% | 41.67% | 16.67% | 24 |
| Qinghai | 32.14% | 28.57% | 39.29% | 28 |
| Shandong | 38.16% | 35.53% | 26.32% | 76 |
| Shanxi | 33.93% | 37.5% | 28.57% | 56 |
| Shaanxi | 33.33% | 33.33% | 33.33% | 45 |
| Shanghai | 39.63% | 32.93% | 27.44% | 164 |
| Sichuan | 51.89% | 27.36% | 20.75% | 106 |
| Tianjin | 26.09% | 43.48% | 30.43% | 69 |
| Tibet | 57.14% | 14.29% | 28.57% | 7 |
| Xinjiang | 21.21% | 36.36% | 42.42% | 33 |
| Yunnan | 40.43% | 40.43% | 19.15% | 47 |
| Zhejiang | 41.86% | 34.11% | 24.03% | 129 |
| Chongqing | 40% | 42.11% | 17.89% | 95 |
